# Comparison of Histologic Characteristics of Chinese Chronic Hepatitis B Patients with Persistently Normal or Mildly Elevated ALT

**DOI:** 10.1371/journal.pone.0080585

**Published:** 2013-11-18

**Authors:** Hong Wang, Li Xue, Rong Yan, Yin Zhou, Ming-Shan Wang, Mei-Juan Cheng, Hai-Jun Huang

**Affiliations:** 1 Department of Infectious Diseases, Zhejiang Provincial People's Hospital, Hangzhou, Zhejiang, China; 2 Department of Pathology, Zhejiang Provincial People's Hospital, Hangzhou, Zhejiang, China; Yonsei University College of Medicine, Korea, Republic Of

## Abstract

Liver disease can develop in chronic hepatitis B (CHB) patients with normal or mildly elevated alanine aminotransferase (ALT) who seldom undergo liver biopsy. We aimed to determine histologic characteristics of a large cohort of Chinese CHB patients undergoing liver biopsy and to evaluate the utility of ALT and HBV DNA values at the time of biopsy in predicting liver disease in this population. This prospective study enrolled 230 treatment-naïve patients with persistently normal or mildly elevated ALT. All patients had a liver biopsy. ALT, aspartate aminotransferase (AST), and HBV DNA levels were some of the other parameters measured. Using Scheuer's classification, significant histology was defined as stage ≧2 fibrosis and/or stage 1 fibrosis plus≧ grade 2 inflammation. Liver disease was observed in 34.4% and 61.8% of patients with normal ALT and mildly elevated ALT, respectively. Patients with mildly elevated ALT levels had significantly more events, including liver disease, elevated AST, and moderate to severe inflammation and liver fibrosis, than patients with normal ALT (all P≤0.005). A total of 107 patients (46.5%) had liver disease and 123 (53.5%) did not. PLT and ALT were significantly associated with liver disease (both P<0.001). Patients with elevated ALT, lower platelet count and HBV DNA < 7 log_10_copies/mL may have histologically significant changes associated with liver disease. Multivariate analysis showed that PLT and HBV DNA levels were significantly associated with liver disease in patients with normal ALT while gender and HBV DNA levels were significantly associated with liver disease in patients with mildly elevated ALT. Assessing liver damage via biopsy in patients with normal or mildly elevated ALT may help to identify those who would benefit from antiviral therapy.

## Introduction

Chronic hepatitis B (CHB) infection is present worldwide with 350 to 400 million individuals reported to be chronically infected [1]. Risk for development of cirrhosis and hepatocellular carcinoma increases significantly when HBV DNA is elevated [2]. HBV infection is common in China, with an estimated ^~^120 million chronically infected persons [3]. In China, most cases of CHB are acquired by vertical transmission (mother-to-child) and patients typically experience a long period of immune tolerance characterized by normal or low levels of the liver enzyme alanine aminotransferase (ALT). Progression of CHB may be influenced by viral load (HBV DNA level), infection with other viruses and probably HBV genotypes [3].

Currently, treatment guidelines for hepatitis B antiviral therapy apply only to patients with an ALT level higher than twice the upper limit of normal (ULN) range [3,4]. Persons with ALT values within the normal range are considered to have “healthy” livers. However, several studies found that moderate inflammation and/or advanced fibrosis can be found in 28%-37% of patients with chronic HBV infection who have persistently normal ALT levels [5-8]. These studies indicate that hepatitis B patients with normal ALT values can have liver disease and may progress to hepatic decompensation. Using ALT values without resorting to liver biopsy to define the inactive carrier state may miss histologically significant disease in a certain proportion of patients. Nevertheless, some investigators have recommended excluding ALT as a criterion for determining which patients are candidates for HBV treatment [9]. However, results of some studies are limited by small samples and the use of liver biopsy only for patients with high serum HBV DNA levels (≥10^5^ copies/ml), raising the possibility of selection bias. Therefore, results of these studies cannot be generalized to all patients with normal ALT levels. Moreover, until now, little information has been available about the liver histology characteristics of CHB patients with slightly elevated ALT.

In the present study, our hypothesis was that a clinically meaningful proportion of chronic HBV infected patients with normal and slightly elevated ALT levels may have liver disease. Therefore, the aims of this study were 1) to determine the incidence of severe liver tissue lesions (pathological changes) in Chinese patients with HBV or HCV infections, 2) to determine the histologic characteristics of a large cohort of CHB patients undergoing liver biopsy, 3) to understand the relationship between ALT and HBV DNA values obtained at the time of biopsy and liver disease, and 4) to investigate other factors that may be associated with liver disease in this population.

## Patients and Methods

### Study design and setting

A prospective study was conducted at Zhe Jiang Provincial People's Hospital between December 1, 2010 and December 1, 2011. The study protocol was reviewed and approved by the ethics committee of Zhe Jiang Provincial People's Hospital. Informed written consent to participate in the study was obtained from each patient.

### Patients

A total of 230 patients with CHB were recruited into the study from the liver center of Zhe Jiang Provincial People's Hospital between December 1, 2010 and December 1, 2011. All consecutive patients who fulfilled the following inclusion criteria were recruited: age≧18, diagnosis of CHB defined by HBsAg positive for more than 6 months; detectable HBV-DNA with a level>10^3^copies/mL; ALT value within normal range (laboratory reference value 50U/L or <2 xULN); no previous or concomitant anti-HBV therapy. Exclusion criteria were: liver comorbidity, including hepatitis delta superinfection, HCV co-infection, chronic alcohol consumption (<30g of pure alcohol per day),Wilson disease, HIV coinfection, or auto-immune hepatitis; present or past evidence of any symptoms related to chronic liver disease, imaging results indicating cirrhosis and evidence of immune suppression. Patients were categorized as persistently normal ALT if they had at least 3 normal ALT values in previous 1 year and no elevated ALT at any time point prior to biopsy; mildly elevated ALT was defined as 1xULN＜ALT levels<2 times ULN. We defined significant liver disease (or significant pathological lesions) as presence of 1) grade 1 inflammation plus stage ≧ 2 fibrosis and / or (2) stage 1 fibrosis plus ≧ grade 2 inflammation. 

### Liver biopsy

All patients received percutaneous liver biopsy guided by ultrasonography. Liver biopsies were performed using 18G biopsy needles. The specimens were fixed, paraffin-embedded, and stained with hematoxylin and eosin (HE stain). A minimum of 1.5cm of liver tissue with at least six portal tracts were required for diagnosis. Histological grading of inflammation (G0 to G3) and staging of the liver fibrosis (S0 to S4) were carried out according to Scheuer's classification. Since we wanted to avoid differences and bias arising from using different examiners, we had all our data examined and evaluated by a single pathologist with over 25 years of experience, who was blinded to the clinical data. Fibrosis was evaluated in all specimens by subjecting them to Masson-Trichrome staining 

### Serum markers

Blood samples of the cohort were obtained on the day before liver biopsy. Biochemical tests for fasting plasma glucose (FPG), total cholesterol (TC), triglycerides (TG), ALT, aspartate aminotransferase (AST), alkaline phosphatase (ALP), GGT, bilirubin, albumin, complete blood count (CBC) were performed in the hospital clinical laboratory using commercially available assays. Hepatitis antibodies HBsAg, HBsAb HBeAg, HBeAb, HBcAb, and anti-HCV were measured using CLIA-approved (Clinical Laboratory Improvement Act) systems compatible with AASLD practice guidelines [4], including anti- HCV kit (Xin Chuan Bioteh Co. Ltd., Dalian，Liaoning, China. The HBsAg, HBeAg and antibody to the hepatitis B e antigen (anti-HBe) were measured using commercially available immunoassays (Abbott Laboratories, Chicago, IL). The serum HBV-DNA levels were measured by a quantitative HBV-DNA PCR kit (Da An Gene Co. Ltd., Xiamen，FuJian, China) with a linear detecting range of 300 to 10^8^ copies/mL and detected with a real-time polymerase chain reaction (PCR) system (ABI7300, Applied Biosystems, Foster City, CA, USA). Normal values for liver enzymes in our clinical laboratory are ALT≤40U/L and AST≤42 U/L in both men and women.

### Statistical analysis

Continuous variables were presented as mean and standard deviation and were compared between groups by Student’s t test. Categorical variables were expressed as count and percentage and compared between groups by Chi-square test. Simple and multiple logistic regression models were performed to identify variables associated with significant liver disease. All significant factors in the univariate analyses were entered into the multivariate models. Potential confounders such as age and gender were automatically adjusted in all multivariate models. All statistical analyses were performed using SAS software version 9.2 (SAS Institute Inc., Cary, NC). A two-sided P value < 0.05 was considered as statistically significant.

## Results

Study subjects (n=230) included 153 (66.5%) males and 77 (33.5%) females with a mean age of 33 ± 9 years. Among these patients, 128 (55.7%) had normal ALT levels and 102 (44.3%) had mildly elevated ALT levels. The baseline characteristics of patients with normal and mildly elevated ALT levels are presented in Table 1. Patients with mildly elevated ALT levels had significantly higher GGT values than patients with normal ALT levels (P=0.001) and also had a significantly greater numbers of events, including liver disease, elevated AST, moderate to severe inflammation, and moderate to severe liver fibrosis, compared with patients with normal ALT levels (all P≤0.005). (Table 1)

**Table 1:** Baseline demographic and clinical characteristics of patients with normal and elevated ALT.

	**Normal ALT (ALT ≤ 40 U/L) (n=128)**	**Mildly elevated ALT (ALT > 40 U/L) (n=102)**	**P-value**
Age (years)^1^	33 ± 10	33 ± 9	0.71
Male ^2^	76 (59.4)	77 (75.5)	0.015*
BMI (kg/m^2^) ^1^	21.0 ± 2.2	21.4 ± 2.2	0.21
Significant liver disease ^2^	44 (34.4)	63 (61.8)	<0.001*
*Clinicalcharacteristics*			
BUN (mmol/L) ^1^	4.8 ± 1.1	4.7 ± 1.2	0.71
Cr (umol/L) ^1^	72.0 ± 17.5	75.0 ± 16.5	0.18
GGT (IU/L) ^1^	27.4 ± 18.3	41.1 ± 35.7	0.001*
AKP (IU/L) ^1^	75.2 ± 22.9	77.5 ± 23.5	0.45
ALB (g/L) ^1^	45.5 ± 2.9	45.1 ± 5.2	0.45
GLB (g/L) ^1^	27.2 ± 4.0	27.0 ± 4.1	0.69
PLT (10^9^/L) ^1^	185.7 ± 50.6	176.0 ± 48.9	0.15
WBC (10^9^/L) ^1^	4.6 ± 0.8	4.7 ± 0.9	0.39
Hb (g/L) ^1^	17.6 ± 21.4	13.9 ± 1.2	0.05
HbeAg+ ^2^	87 (68.0)	71 (69.6)	0.90
AST (IU/L) ^2^			<0.001*
≤ 42	123 (96.1)	52 (51.0)	
> 42	5 (3.9)	50 (49.0)	
HBV DNA(log_10_copies/mL) ^2^			0.48
<7	70 (55.6)	51 (50.0)	
≥ 7	56 (44.4)	51 (50.0)	
Grading of inflammation^2^			<0.001*
Mild (G0 and G1)	96 (75.0)	50 (49.0)	
Moderate to severe (G2 and G3)	32 (25.0)	52 (51.0)	
Stage of liver fibrosis^2^			0.005*
Mild (S0 and S1)	104 (81.3)	65 (63.7)	
Moderate to severe (S2 to S4)	24 (18.8)	37 (36.3)	

BMI, body mass index; AKP, alkaline phosphatase; GGT, γ-glutamyltransferase; ALB, albumin; ALT, alanine aminotransferase; AST, aspartate aminotransferase; BUN, blood urea nitrogen; CR, creatinine; GLB, globulin; WBC, white blood cell count; PLT, Platelet.;HbeAg, hepatitis B e antigen;Hb,hemoglobin.

^1^ Continuous variables are presented as mean and standard deviation as examined by Student’s t test ^2^;categorical variables are expressed as counts and percentages as examined by Chi-square test.

* P <0.05 indicates a significant difference between normal and elevated ALT patients group.

A total of 107 of our study patients (46.5%) had liver disease and 123 (53.5%) did not. The baseline demographic and clinical characteristics of patients with and without liver disease are presented in Table 2. Patients with liver disease had significantly higher GGT values (P=0.001) and lower ALB and PLT values (both P≤0.015) compared to patients without liver disease. The proportions of patients with elevated ALT, elevated AST, HBV DNA < 7 x10^4^copies/ml, moderate to severe inflammation, and moderate to severe liver fibrosis were significantly greater among patients with liver disease compared to patients without liver disease (all P≤0.001). (Table 2).

**Table 2:** Demographic and clinical characteristics of patients with and without significant liver disease.

	**No significant liver disease (n=123)**	**Significant liver disease (n=107)**	**P-value**
Age (years) ^1^	32 ± 9	34 ± 9	0.06
Male^2^	84 (68.3)	69 (64.5)	0.64
BMI (kg/m^2^) ^1^	21.1 ± 2.2	21.3 ± 2.2	0.42
*Clinicalcharacteristics*			
BUN (mmol/L) ^1^	4.7 ± 1.1	4.8 ± 1.2	0.60
Cr (umol/L) ^1^	73.0 ± 17.3	73.8 ± 16.9	0.73
GGT (IU/L) ^1^	27.5 ± 17.7	40.5 ± 35.7	0.001*
AKP (IU/L) ^1^	73.7 ± 22.2	79.2 ± 24.0	0.07
ALB (g/L) ^1^	46.0 ± 2.8	44.6 ± 5.1	0.015*
GLB (g/L) ^1^	26.7 ± 3.8	27.5 ± 4.3	0.11
PLT (10^9^/L) ^1^	193.0 ± 48.0	168.1 ± 49.1	<0.001*
WBC (10^9^/L) ^1^	4.6 ± 0.9	4.6 ± 0.9	0.71
Hb (g/L) ^1^	15.0 ± 13.3	17.1 ± 18.7	0.34
HbeAg+^2^	90 (73.2)	68 (63.6)	0.15
ALT (IU/L)^2^			<0.001*
≤ 40	84 (68.3)	44 (41.1)	
> 40	39 (31.7)	63 (58.9)	
AST (IU/L)^2^			<0.001*
≤ 42	109 (88.6)	66 (61.7)	
> 42	14 (11.4)	41 (38.3)	
HBV DNA(log_10_copies/mL)^2^			0.001*
<7	52 (42.6)	69 (65.1)	
≥ 7	70 (57.4)	37 (34.9)	
Grading of inflammation^2^			<0.001*
Mild (G0 and G1)	123 (100.0)	23 (21.5)	
Moderate to severe (G2 and G3)	0 (0.0)	84 (78.5)	
Stage of liver fibrosis^2^			<0.001*
Mild (S0 and S1)	123 (100.0)	46 (43.0)	
Moderate to severe (S2 to S4)	0 (0.0)	61 (57.0)	

BMI, body mass index; AKP, alkaline phosphatase; GGT, γ-glutamyltransferase; ALB, albumin; ALT, alanine aminotransferase; AST, aspartate aminotransferase; BUN, blood urea nitrogen; CR, creatinine; GLB, globulin; WBC, white blood cell count; PLT, Platelet;HbeAg, hepatitis B e antigen; Hb,hemoglobin.

^1^ Continuous variables are presented as mean and standard deviation as examined by Student’s t test ^2^;categorical variables are expressed as counts and percentages as examined by Chi-square test.

* P <0.05 indicates a significant difference between patients with and without significant liver disease.

Variables associated with the presence of liver disease are identified in Table 3. Results of univariate logistic regression analysis showed that GGT, ALB, PLT, ALT, AST and HBV DNA were all significantly associated with the presence of liver disease (all P≤0.014). After adjustments for age, gender, GGT, ALB, ALT, AST and HBV DNA, multivariate logistic regression analysis revealed that PLT was significantly associated with the presence of liver disease (OR=0.99, 95% CI=0.98-1.00, P=0.007). After adjustments for age, gender, GGT, ALB, PLT, AST and HBV DNA, elevated ALT level was positively associated with the presence of liver disease (OR=2.47, 95% CI=1.20-5.08, P=0.014). After adjustments for age, gender, GGT, ALB, PLT, ALT and AST, multivariate logistic regression analysis revealed that HBV DNA was significantly associated with the presence of liver disease (OR=0.40, 95% CI=0.21-0.77, P=0.006). (Table 3).

**Table 3:** Univariate and multivariate regression analysis of confounding variables of significant liver disease in all patients.

	**Univariate**		**Multivariate**	
	**Crude OR (95%CI)**	**P-value**	**Adjusted OR (95%CI)**	**P-value**
Age (years)	1.03 (1.00,1.06)	0.06	1.00 (0.97,1.04)	0.90
Male	0.84 (0.49,1.46)	0.54	0.58 (0.29,1.16)	0.12
BMI (kg/m^2^)	1.05 (0.93,1.18)	0.41		
*Clinicalcharacteristics*				
BUN (mmol/L)	1.06 (0.85,1.33)	0.60		
Cr (umol/L)	11.00 (0.99,1.02)	0.72		
GGT (IU/L)	1.02 (1.01,1.03)	0.001*	1.01 (1.00,1.03)	0.06
AKP (IU/L)	1.01 (11.00,1.02)	0.08		
ALB (g/L)	0.90 (0.82,0.98)	0.014*	0.93 (0.85,1.02)	0.14
GLB (g/L)	1.05 (0.99,1.13)	0.11		
PLT (10^9^/L)	0.99 (0.98,0.99)	<0.001*	0.99 (0.98,1.00)	0.007*
WBC (10^9^/L)	0.94 (0.70,1.27)	0.71		
Hb (g/L)	1.01 (0.99,1.03)	0.35		
HbeAg+	0.64 (0.37,1.12)	0.12		
ALT (IU/L)				
≤ 40	1		1	
> 40	3.08 (1.80,5.30)	<0.001*	2.47 (1.20,5.08)	0.014*
AST (IU/L)				
≤ 42	1		1	
> 42	4.84 (2.45,9.54)	<0.001*	2.20 (0.94,5.10)	0.07
HBV DNA(log_10_copies/mL)				
< 7	1		1	
≥ 7	0.40 (0.23,0.68)	0.001*	0.40 (0.21,0.77)	0.006*
Grading of inflammation^2^				
Mild (G0 and G1)	NA			
Moderate to severe (G2 and G3)	NA			
Stage of liver fibrosis^2^				
Mild (S0 and S1)	NA			
Moderate to severe (S2 to S4)	NA			

BMI, body mass index; AKP, alkaline phosphatase; GGT, γ-glutamyltransferase; ALB, albumin; ALT, alanine aminotransferase; AST, aspartate aminotransferase; BUN, blood urea nitrogen; CR, creatinine; GLB, globulin; WBC, white blood cell count; PLT, Platelet;HbeAg, hepatitis B e antigen; Hb,hemoglobin.

2 categorical variables are expressed as counts and percentages as examined by Chi-square test

NA, not applicable; * P<0.05 indicated as a potential confounder.

For patients with normal ALT levels, both univariate and multivariate logistic regression analysis revealed that only PLT and HBV DNA were significantly associated with the presence of liver disease (all P≤0.045) (Table 4). 

**Table 4:** Univariate and multivariate regression analysis of confounding variables of significant liver disease.

	***NormalALT***					***Mildly elevated ALT***				
	**Univariate**			**Multivariate**		**Univariate**			**Multivariate**	
	**Crude OR (95%CI)**	**P-value**		**Adjusted OR (95%CI)**	**P-value**	**Crude OR(95%CI)**	**P-value**		**Crude OR (95%CI)**	**P-value**
Age (years)	1.02 (0.99,1.06)	0.22		1.00 (0.95,1.04)	0.83	1.04 (0.99,1.10)	0.08		1.01 (0.95,1.07)	0.83
Male	0.85 (0.41,1.78)	0.67		0.71 (0.32,1.56)	0.39	0.42 (0.15,1.17)	0.10		0.26 (0.07,0.99)	0.048*
BMI (kg/m^2^)	1.07 (0.901.26)	0.44				0.99 (0.83,1.19)	0.92			
*Clinicalcharacteristics*										
BUN (mmol/L)	1.10 (0.80,1.51)	0.57				1.06 (0.75,1.5)	0.73			
Cr (umol/L)	1.00 (0.98,1.03)	0.69				0.99 (0.97,1.02)	0.63			
GGT (IU/L)	1.00 (0.98,1.02)	0.72				1.03 (1.01,1.05)	0.011*		1.03 (1.00,1.06)	0.031*
AKP (IU/L)	1.01 (1.00,1.03)	0.09				1.00 (0.99,1.02)	0.58			
ALB (g/L)	0.91 (0.80,1.03)	0.14				0.87 (0.75,1.00)	0.044*		0.92 (0.78,1.07)	0.27
GLB (g/L)	1.03 (0.94,1.13)	0.54				1.11 (1.00,1.23)	0.05			
PLT (10^9^/L)	0.99 (0.98,1.00)	0.007*		0.99 (0.98,1.00)	0.020*	0.99 (0.98,1.00)	0.034*		0.99 (0.98,1.00)	0.08
WBC (10^9^/L)	0.96 (0.61,1.49)	0.84				0.86 (0.57,1.32)	0.49			
Hb (g/L)	1.01 (0.99,1.03)	0.17				1.20 (0.85,1.69)	0.31			
HbeAg+	0.64 (0.29,1.37)	0.25				0.56 (0.23,1.39)	0.21			
AST (IU/L)										
≤ 42	1					1			1	
> 42	1.29 (0.21,8.00)	0.79				4.14 (1.74,9.81)	0.001*		2.60 (0.96,7.03)	0.06
HBV DNA(log_10_copies/mL)										
< 7	1					1				
≥ 7	0.40 (0.18,0.88)	0.022*		0.42 (0.18,0.98)	0.045*	0.27 (0.12,0.64)	0.003*		0.30 (0.11,0.85)	0.024*
Grading of inflammation^2^										
Mild (G0 and G1)	NA					NA				
Moderate to severe (G2 and G3)	NA					NA				
Stage of liver fibrosis^2^										
Mild (S0 and S1)	NA					NA				
Moderate to severe (S2 to S4)	NA					NA				

BMI, body mass index; AKP, alkaline phosphatase; GGT, γ-glutamyltransferase; ALB, albumin; ALT, alanine aminotransferase; AST, aspartate aminotransferase; BUN, blood urea nitrogen; CR, creatinine; GLB, globulin; WBC, white blood cell count; PLT, Platelet; HbeAg, hepatitis B e antigen; Hb,hemoglobin.

2 categorical variables are expressed as counts and percentages as examined by Chi-square test

NA, not applicable; * P<0.05 indicated as a potential confounder.

For patients with mildly elevated ALT levels, results of univariate logistic regression analysis showed that GGT, ALB, PLT, elevated AST levels and HBV DNA were significantly associated with the presence of liver disease (all P≤0.044). After adjustments for age and gender to control for effects of potential confounders, results of multivariate analysis showed that gender (OR=0.26, 95% CI=0.07-0.99, P=0.048), GGT (OR=1.03, 95% CI=1.00-1.06, P=0.031) and HBV DNA (≥ 7 x10^4^copies/ml) (OR=0.30, 95% CI=0.11-0.85, P=0.024) were significantly associated with the presence of liver disease. (Table 4)

## Discussion

Results of the present study revealed that nearly half (46.5%) of CHB patients with normal and mildly elevated ALT values had liver disease. Among all patients studied, those with elevated ALT levels had a significantly greater number of liver-related events than those with normal ALT values, including the presence of liver disease, elevated AST, and moderate to severe inflammation and liver fibrosis. In addition, GGT, ALB, PLT, ALT and AST were all significantly associated with the presence of liver disease. Multivariate logistic regression analysis revealed that patients with elevated ALT who had liver disease had significantly higher GGT values and lower ALB and PLT counts than patients without liver disease. Multivariate analysis also showed that HBV DNA was significantly associated with liver disease in patients with normal ALT levels as well as in patients with mildly elevated ALT levels. 

Our present findings are consistent with previous reports that found liver disease in patients with normal or slightly elevated ALT values [5-8]. Lai et al. [6] found that 37% of CHB patients with persistently normal ALT levels had a significant amount of fibrosis and inflammation on liver biopsy and those investigators recommended considering liver biopsy in patients over age 40 with high normal ALT. Although a European study found that patients with CHB infection frequently had extensive liver fibrosis or cirrhosis, no differences were seen in the degree of fibrosis or frequency of cirrhosis between patients with normal vs. elevated transaminases; the most important factor in that study was age over 40 years [10]. However, a retrospective analysis of 743 CHB patients whose data were collected over a ten-year period, reported that liver disease was rare in chronic HBV-infected patients with persistently normal ALT, but the authors emphasized that the liver histology in patients with normal ALT values could not be generalized to others with normal ALT who had not been biopsied [11]. The instance of “rare” in that study may possibly be explained by age differences and immune status as noted in the study by Lai et al. [6], in which significant histological findings were obtained in a minority of younger CHB patients who were immune tolerant but the majority of significant histological findings were in patients with high normal ALT. We believe that our detailed histological evaluations are an important merit of this study.

Another study in China concluded that many CHB patients with ALT twice the ULN had significant amounts of liver inflammation or fibrosis and that liver biopsy was essential to determine who should receive antiviral therapy [7]. As in the present study, nearly 50% of patients with ALT within the normal range had liver disease but the frequency of liver damage was similar across different levels of ALT. To some authors, this suggests that finding significant levels of fibrosis in a high percentage of patients with normal ALT represents a poor response to antiviral treatment rather than a correlation of ALT levels with hepatic fibrosis [12]. On the other hand, data from a recent large population histology study showed poor correlation of elevated ALT and liver disease in HBeAg-positive and HBeAg–negative patients [13]. In addition, Prati et al. [14] showed that ALT was not an accurate predictor of liver histology, noting that ALT activity failed to identify many patients with hepatic injury. That study found that increased ALT activity was independently associated with BMI and abnormal lipid parameters and that updating the upper limits of normal showed superior sensitivity in detecting HCV viremia among over 6000 blood donors with anti-HCV antibodies. The authors suggested revising the standards of normal ALT at that time (2002), which were based on populations with subclinical liver disease. Today’s standards must meet laboratory performance requirements of the AASLD, which note that ALT upper reference limits increase from childhood through adulthood, especially in males, and that the upper limits will be about 10% higher in men aged 40 years compared to men aged 25 years [4]. Using data from patients with stable ALT, an error factor of less than 10% is required at upper reference limits to identify candidates for antiviral therapy. However, current methods do not guarantee this. Therefore, new standards are recommended for ALT, which may require developing new methods. In fact, the results of Wang et al. [15] highlight the need to apply stringent definitions of normal serum ALT when making clinical decisions for CHB patients for whom older age and lower serum HBV DNA levels may predict a significant amount of hepatic fibrosis on biopsy.

Serum HBV DNA levels were previously shown to correlate with the development of hepatocellular carcinoma and cirrhosis independent of serum ALT level, HBV genotype and HBeAg status [2]. In the present study, we showed that HBV DNA levels (> 7 x10^4^copies/ml) were significantly associated with the presence of liver disease in patients with normal ALT as well as in patients with mildly elevated ALT. Although HBV DNA levels are negatively associated with age, the longer duration of HBV infection (persistent disease with viremia), is associated with fibrosis risk [2]. It is interesting to note that chronic HBV infection is dynamic, evolving from an early immunotolerant phase to a later immunoactive phase [16] and decreased serum HBV DNA may be an important marker of increasing immune system activity [17]. In patients > 40 years, the longer duration of increasing immune system activity results in a higher risk of liver inflammation and subsequent liver fibrosis. 

When classifying the clinical phases of HBV, immunotolerant patients are commonly defined as young, with active viral replication (usually HBV DNA>10^7^ copies/ml), HBeAg positive and normal ALT [18]. However, since the normal ALT value is usually defined as 40 U/L, some patients with meaningful liver lesions are misclassified as being in the tolerant phase. Therefore, it has been suggested that lower “normal” ALT values (30 U/L for males and 19 U/L for females) would be more useful in distinguishing between the states of immune tolerance and immune clearance [12]. Although it can be argued that there could be a certain bias in our study arising from the relatively young age of the patients, our experimental groups were within the same age distribution. It is also important to note that our patients were recruited based on their ALT levels, and not based on histological findings. 

Results of the present study did not identify HBeAg status as a significant predictor of histology outcomes for patients with normal ALT or with mildly elevated ALT. This lack of association between HBeAg status and histological outcome may be due to the short observation period. However, similar results were also observed in other studies of patients with normal ALT levels [6,7], and HBeAg-negative patients with persistently normal ALT levels were rare with histological significant changes within liver disease [19]. However, Wong et al. reported that *positive* HBeAg in patients with older age and higher ALT levels may indicate abortive immune clearance, yielding a higher risk of advanced fibrosis [20]. Puoti et al. [21] described two different subsets of patients with normal ALT levels and positive HBsAg: inactive HBV carriers and patients with transient virological and biochemical remission. Management of these patients must include evaluation of ALT values and HBV DNA at least every six months and observation of ALT flares may suggest liver biopsy and antiviral treatment.

Also in the present study, we found no significant difference in baseline platelet counts between patients in the normal ALT group and those in the slightly elevated ALT group. However, patients with significant liver disease had lower platelet counts compared to patients without significant liver disease..In fact, in patients with normal ALT levels, both univariate and multivariate logistic regression analysis revealed that *only* the platelet count was significantly associated with the presence of liver disease, while in CHB patients with elevated ALT, GGT and platelet count were *both* significantly associated with the presence of liver disease. Previous studies also have found that decreased platelet counts were associated with progression of liver fibrosis [12,22,23], which is due largely to decreased production of thrombopoietin by hepatocytes [22]. The high mean platelet counts in both groups of patients could be a reflection of the age of the patients in this study.

The appropriate approach to patients with active viremia and normal ALT has been described as individualized care and performance of a liver biopsy [24]. In patients with normal ALT, the decision to perform a liver biopsy should ideally balance the cost and risks of liver biopsy against the chance of not identifying patients whose disease will progress without treatment. Previous studies have demonstrated that non-invasive bio-markers such as APRI [25] and FIB-4 [26] using standard biochemical laboratory values are able to provide simple and inexpensive measures of significant histology for patients with normal ALT, even though these tests were shown to have a negative predictive value for significant histology. It is reasonable then to initially evaluate all patients with normal ALT by such non-invasive methods, including AST levels, and then to decide which ones may require liver biopsy. One Chinese study identified significant histopathologic findings in patients with persistently normal ALT correlated with age and ALT level, with data supporting liver biopsy in CHB patients over age 40 years when the ALT value is 0.5-1.0 x ULN [27]. In the present study, 61.8% of patients with mildly elevated ALT (1×ULN<ALT<2×ULN) had significant histology, and subgroup analysis revealed a higher prevalence of significant histology in patients with mildly elevated ALT than in those with normal ALT. This appears to suggest that liver biopsy is necessary for almost all patients with mildly elevated ALT, not just for patients older than 40 years as AASLD guidelines and the results of some studies [27] suggest. This practice would certainly help to initiate antiviral treatment early in patients who require it. Our data suggest a greater role for liver biopsy in patients with mildly elevated ALT. 

This study has certain limitations. Patients who did not agree to a liver biopsy were excluded and many CHB patients with persistently normal or mildly elevated ALT did not undergo a liver biopsy, thereby generating a potential selection bias. Another limitation is that fibrosis and inflammation were evaluated using only the Scheuer classification system, and the Knodell-Ishak score was not assessed. Other important limitations of this study are that our histological findings were based on only 6 portal tracts and the time for patients were categorized as persistently normal ALT within previous 1 year. Additionally, HBV genotypes were not assessed because genotyping is not routine clinical practice in our hospital. Therefore, we cannot rule out genetic influences such as genotype C HBV, which has been consistently shown to be associated with active liver disease compared to genotype B HBV in Asian patients [28,29]. Further studies of larger cohorts are needed, including patients with low HBV DNA levels and normal and mildly elevated ALT, to assess the true distribution of significant histological disease among CHB patients. Studies investigating novel biomarkers with high positive predictive value for liver injury and fibrosis should also include this patient population.

In conclusion, liver disease is found in a significant proportion of Chinese patients with normal and mildly elevated ALT. Patients over age 40 years with elevated ALT, high GGT and low platelet count may have histologically significant changes within liver disease. The use of liver biopsy to assess liver damage in such patients may help to identify those who would benefit from antiviral therapy. 
